# Effective evaluation of HGcnMLP method for markerless 3D pose estimation of musculoskeletal diseases patients based on smartphone monocular video

**DOI:** 10.3389/fbioe.2023.1335251

**Published:** 2024-01-09

**Authors:** Rui Hu, Yanan Diao, Yingchi Wang, Gaoqiang Li, Rong He, Yunkun Ning, Nan Lou, Guanglin Li, Guoru Zhao

**Affiliations:** ^1^ CAS Key Laboratory of Human-Machine Intelligence-Synergy Systems, Research Center for Neural Engineering, Shenzhen Institute of Advanced Technology, Chinese Academy of Sciences, Shenzhen, China; ^2^ Shenzhen College of Advanced Technology, University of Chinese Academy of Sciences, Shenzhen, China; ^3^ Department of Orthopedic and Rehabilitation Center, University of Hong Kong–Shenzhen Hospital, Shenzhen, China

**Keywords:** markerless pose estimation, rehabilitation assessment, high-resolution graph convolutional multilayer perception (HGcnMLP), smartphone monocular video, musculoskeletal disorders

## Abstract

Markerless pose estimation based on computer vision provides a simpler and cheaper alternative to human motion capture, with great potential for clinical diagnosis and remote rehabilitation assessment. Currently, the markerless 3D pose estimation is mainly based on multi-view technology, while the more promising single-view technology has defects such as low accuracy and reliability, which seriously limits clinical application. This study proposes a high-resolution graph convolutional multilayer perception (HGcnMLP) human 3D pose estimation framework for smartphone monocular videos and estimates 15 healthy adults and 12 patients with musculoskeletal disorders (sarcopenia and osteoarthritis) gait spatiotemporal, knee angle, and center-of-mass (COM) velocity parameters, etc., and compared with the VICON gold standard system. The results show that most of the calculated parameters have excellent reliability (VICON, ICC (2, k): 0.853–0.982; Phone, ICC (2, k): 0.839–0.975) and validity (Pearson r: 0.808–0.978, p < 0.05). In addition, the proposed system can better evaluate human gait balance ability, and the K-means++ clustering algorithm can successfully distinguish patients into different recovery level groups. This study verifies the potential of a single smartphone video for 3D human pose estimation for rehabilitation auxiliary diagnosis and balance level recognition, and is an effective attempt at the clinical application of emerging computer vision technology. In the future, it is hoped that the corresponding smartphone program will be developed to provide a low-cost, effective, and simple new tool for remote monitoring and rehabilitation assessment of patients.

## 1 Introduction

Human motion capture systems can quantify and analyze complex sports injuries and balance abilities and have been widely used as a basic technology in biomechanics research (Scott et al., 2022). The parameters captured by the system can provide objective and reliable information for clinical decision-making, for example, auxiliary diagnosis and rehabilitation assessment of musculoskeletal diseases (Aleixo et al., 2019; Kim et al., 2023). Currently, the gold standard for human motion capture is marker-based multi-camera and reflective systems. For example, the VICON motion capture system has the advantages of high precision and stability (Aoyagi et al., 2022). Some studies have demonstrated that marker systems are also proficient in identifying other motor tasks, such as upper limb or trunk balance control (Mailleux et al., 2017; Haberfehlner et al., 2020). However, marker-based motion capture systems also have obvious limitations: a) the system requires dedicated hardware and software; b) the optical markers need to be placed very accurately, which is unfriendly to patients; c) the system is limited to a closed environment and required a professional physical therapist for calibration (Biasi et al., 2015), the above disadvantages limit the promotion of systematic clinical balance estimation and home telerehabilitation.

Markerless pose estimation algorithms based on computer vision and deep neural networks offer a simpler and cheaper alternative to human motion capture (Moro et al., 2022). Compared with marker-based motion capture methods, the markerless method is simple and does not require reflective markers to be placed on the patient, thereby reducing clinician workload and improving efficiency (Avogaro et al., 2023). Furthermore, the data can be easily acquired through common household devices (e.g., webcams, smartphones), which offers the potential to deploy such systems with minimal cost (Stenum et al., 2021a). A markerless system typically consists of four main components: the camera system, the 3D skeletal model, the image features, and algorithms for determining the model shape, pose, and position (Colyer et al., 2018). Among them, the pose estimation algorithm is mainly based on the marked video data to train the neural network, then estimate the human body pose when inputting the user image or video into the trained network, such as the joint center and bones, and finally obtain the pose information (Nakano et al., 2020). Several studies have shown that markerless human 3D pose estimation techniques have great potential for motion capture and remote rehabilitation assessment (Hellsten et al., 2021).

The deep learning-based markerless approach starts with 2D pose estimation, which automatically estimates human joint centers from 2D RGB images and outputs 2D coordinates (Wei et al., 2016; Papandreou et al., 2018). Kidziński et al. detected key points from 2D images for gait analysis, then extracted joint angles, and analyzed their changes during the gait period (Kidziński et al., 2020). Moro et al. investigated the gait patterns of 10 stroke patients and performed quantitative balance analysis, but the 2D characteristics of the images limited the analysis to a subset of elevation and spatiotemporal parameters (Moro et al., 2020). The Carnegie Mellon University research team released the OpenPose processing framework, which can identify multiple human skeletons in the same scene and become the most popular open-source pose estimation technology (Cao et al., 2017). Yagi et al. used OpenPose to detect multiple individuals and joints in the image to estimate gait, step length, step width, walking speed, cadence and compared it with a multiple infrared camera motion capture (OptiTrack) system (Yagi et al., 2020). Stenum et al. used OpenPose to compare spatiotemporal and sagittal motion gait parameters of healthy adults with optical marker-based features captured during walking (Stenum et al., 2021b). Recently, some researchers have detected 3D skeletons from images based on 2D human pose detectors by directly using image features (Martinez et al., 2017; Moreno-Noguer, 2017; Zhou et al., 2018). Martinez et al. used a relatively simple deep feed-forward network to efficiently lift 2D poses to 3D poses (Martinez et al., 2017). Nakano et al. compared the joint positions estimated by the OpenPose-based 3D markerless motion capture technique with the results of motion capture recordings (Nakano et al., 2020). For clinical diagnosis and posture balance assessment, 3D human motion capture can obtain more dimensional posture information (e.g., center of mass, step width, step length, etc.) and provide a more reliable basis for clinical decision-making, which only relies on 2D key points is difficult to achieve. Therefore, even though currently extracting 2D key points is more accurate than 3D models (many 3D key point extraction relies on the input of 2D key points), the latter shows more potential clinical biomechanical applications than 2D models (Hellsten et al., 2021).

There are two main categories of 3D human pose estimation: the first is to directly regress 3D human joints from RGB images (Pavlakos et al., 2017); the second is the 2D-to-3D pose enhancement method (Li and Lee, 2019; Gong et al., 2021), which uses 2D pose detection as input, and then design a 2D to 3D lifting network to finally achieve optimal performance. The second method has become a mainstream method due to its efficiency and effectiveness. When identifying 2D key points, most existing methods take input according to network transfer, and go from high-resolution to low-resolution sub-networks in a concatenated manner (Sun et al., 2019), and finally increase the resolution, which generally reduce resolution and affect accuracy (Newell et al., 2016; Xiao et al., 2018). Therefore, a novel high-resolution network (HRNet) architecture is proposed and is able to maintain high-resolution representation throughout the process (Nibali et al., 2019). On the other hand, during the 2D to 3D pose conversion process, multiple 3D joint positions may correspond to the same 2D projection in the image, which will affect the accuracy of the results. The graph convolutional networks (GCN) have been intensively used to solve the problem of 2D to 3D pose enhancement (Wang et al., 2020). However, although GCN-based methods can effectively aggregate adjacent nodes to extract local features, they ignore the global information between body joints, which is crucial for overall body pose (Cai et al., 2019). Modern multi-layer perceptron (MLP) with global views have shown great power on various vision tasks and are used to extract joint global information (Shavit et al., 2021). Nevertheless, there are still two flaws: 1) the connection between all relevant nodes is simple, and the model is inefficient in terms of graph-structured data; 2) the model is not good at capturing local interactions due to the lack of fine design between adjacent joints. To overcome the above limitations, this study intends to adopt the GraphMLP framework, which is a global-local-graph unified architecture that establishes strong collaboration between modern MLP and GCN for learning better skeletal representations (Zhao et al., 2023). In addition, the GraphMLP framework has the advantages of being lightweight and low computational cost, making it very suitable for clinical needs of low-cost, simple, and effective evaluation (Li et al., 2022).

Additionally, the markerless pose estimation systems include multi-view and single-view techniques (Desmarais et al., 2021). In general, multi-view systems are more accurate and popular for human 3D pose estimation, but they require hardware and synchronization between different cameras. From a physical therapy perspective, the most promising technique is single-view 3D markerless pose estimation, which enables advanced motion analysis of the human body while requiring only a single camera and computing device (Hellsten et al., 2021). Pavlakos and Bogo et al., 2016 estimated 3D human pose from a single image and pointed out that this method provides an attractive solution for directly predicting 3D pose (Pavlakos et al., 2018). Colyer et al. pointed out that it is unclear whether markerless 3D motion capture is suitable for telerehabilitation of human motion studies due to low accuracy (Needham et al., 2021). Kim et al., 2023 performed plantar pressure and 2D pose estimation on patients with musculoskeletal diseases through smart insoles and mobile phones, and the results showed that the extracted parameters had the potential to identify sarcopenia, but lacked 3D pose and reliability analysis. Aoyagi et al., 2022 tracked 3D human motion in real-time based on smartphone monocular video, but the accuracy rate needs to be further improved. To our knowledge, 3D pose estimation and simultaneous quantitative analysis of balance ability in patients with musculoskeletal diseases based on a single smartphone monocular video has not been reported.

In summary, the purpose of this study is to explore the potential of markerless 3D human pose estimation based on smartphone monocular video for gait balance and rehabilitation assessment. The main goals include: 1) Based on the 2D pose estimation high-resolution network framework, combining the advantages of modern MLP and GCN networks to construct a markerless 3D pose estimation model, which can capture local and global interaction information; 2) Based on the smartphone monocular video 3D human pose estimation algorithm, extract the parameters of healthy subjects and patients with musculoskeletal diseases (sarcopenia and osteoarthritis), and verify the reliability and effectiveness; 3) Extract sensitivity indicators for balance evaluation between healthy adults and patients, identify high-low levels of patient recovery progress, and verify the performance of the system in practical clinical applications. Overall, we hope that the proposed markerless human 3D pose estimation algorithm can provide a simple, effective, and easy-to-operate new tool for patient monitoring and rehabilitation assessment.

## 2 Experiment

**FIGURE 1:**
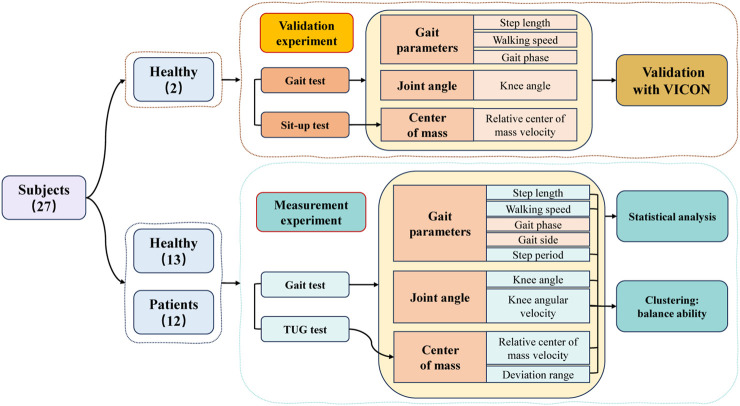
The flowchart and objectives of this research.

The flowchart of this study, as illustrated in Figure 1, begins with a validation experiment to ascertain the reliability and effectiveness of the proposed method. Subsequently, measurement experiment are conducted on healthy adults and patients, followed by a thorough analysis of their respective datasets. Finally, employing distinctive features displaying significant differences, the data from healthy adults and patients undergo cluster analysis to achieve a graded stratification of their functional capabilities.

### 2.1 Participants

A total of 27 participants were recruited for this study, comprising 12 patients and 15 healthy adults. All patients were recruited from the University of Hong Kong Shenzhen Hospital, and inclusion criteria were as follows: 1) The patient is clinically diagnosed with musculoskeletal diseases (sarcopenia and osteoarthritis); 2) All patients have gait instability or balance dysfunction; 3) Patients were capable of walking independently or with the use of assistive devices; 4) Patients had no cognitive impairments and were capable of completing all experiments independently. Healthy participants were laboratory students aged between 20 and 35 years. Table 1 presents the demographic characteristics of the participants. Before the experiment, professional physical therapists will evaluate the participants’ balance ability through clinical scales and gait tests. All participants signed informed consent forms, and all human-related experiments received ethical approval from the Medical Research Ethics Committee of the Shenzhen Institutes of Advanced Technology, Chinese Academy of Sciences. SIAT-IRB-230915-H0671.

**TABLE 1:** Demographic characteristics of patients and healthy adults.

Cohort information	Mean (Standard deviation)				p
	Healthy adults		Patients		
Gender	Female	Male	Female	Male	—
Number	8	7	8	4	—
Age (years)	23.0 (4.87)	24.9 (1.86)	67.9 (5.89)	68.0 (1.41)	<0.01
Height (cm)	161.8 (4.03)	178.9 (7.36)	159.0 (4.47)	164.5 (4.93)	0.04
Weight (kg)	55.5 (7.37)	74.9 (4.10)	66.1 (8.76)	70.3 (4.03)	0.25

### 2.2 Experimental setup

#### 2.2.1 Measurement equipment and facilities

**FIGURE 2:**
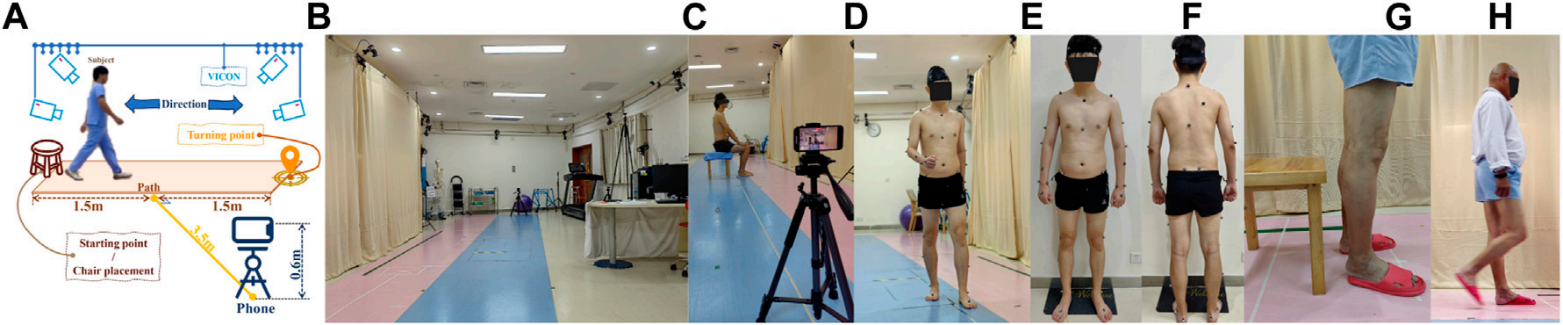
Experimental Setup, **(A)** Site setup, **(B)** Experimental site, **(C)** Sit-up test (Validation experiment), **(D)** Gait test (Validation experiment), **(E)** Markers (Front), **(F)** Markers (Back), **(G)** TUG test (Measurement experiment), and **(H)** Gait test (Measurement experiment).

Video data was collected using an iPhone 14 smartphone (Apple Inc., Cupertino, CA, United States) with an original image resolution of 1920 × 1080 pixels and a frame rate of 30 Hz (auto-focus state). The smartphone, measuring 146.7 × 71.5 × 7.8 mm, was positioned vertically at a distance of 3.5 m from the walking path and at a height of 0.6 m (Figure 2A). The measurement path was set at a length of 3 m and was within the measurement range of the Vicon equipment (Figure 2B).

#### 2.2.2 Vicon

The VICON system consists of 12 infrared cameras and 2 video cameras, which are used to capture human motion data during gait and serve as the gold standard. Before the experimental data collection, a professional physical therapist placed 39 reflective markers on the bone landmarks of the participants, including the head (4), trunk (5), upper limbs (14), and lower limbs (12) (Figures 2E,F). After completing the reflective markers, participants need to go to the center of the venue for posture calibration to prevent the loss of data. The size of the VICON system site is about 15 m × 8 m, which provides enough space for the execution of gait experiments.

### 2.3 Data acquisition

Participants were requested to wear minimal clothing during the experiments. Two participants (healthy adults) were involved in the validation experiment, during which video data were simultaneously collected by both the VICON system and the smartphone. In the measurement experiment, video data were exclusively captured by the smartphone, involving 25 participants (13 healthy individuals and 12 patients). Actions designed in validation experiments are the basic building elements of actions designed in measurement experiments.

#### 2.3.1 Validation experiment

During the gait test (Figure 2D), participants were instructed to walk at their usual pace along a 3-m-long corridor from the starting point to the turning point, where they would then turn around and walk back to the starting point. (Figure 2A).

In the sit-up test (Figure 2C), a chair was positioned at the midpoint of the corridor. Participants were required to sit perpendicular to the smartphone’s camera at a 90° angle. Once the sit-up test commenced, participants were asked to stand up and maintain a stationary position for 1 s, after which they were to sit back down and remain still for an additional 1 s. Both the gait test and the sit-up test were repeated twice.

#### 2.3.2 Measurement experiment

During the gait test (Figure 2H), participants were instructed to walk at their customary pace along a 3-m-long corridor, starting from the starting point, proceeding to the turning point, and then turning around to walk back to the starting point.

For the Time Up and Go test (TUG test) (Figure 2G), a chair was positioned at the starting point. Participants were required to sit in a chair. Once the test commenced, participants were instructed to walk at their usual pace to the turning point 3 m away, then turn around and walk back to the starting point before sitting down. Both the gait test and the TUG test were repeated three times.

## 3 Method

### 3.1 Data preprocessing

**FIGURE 3:**
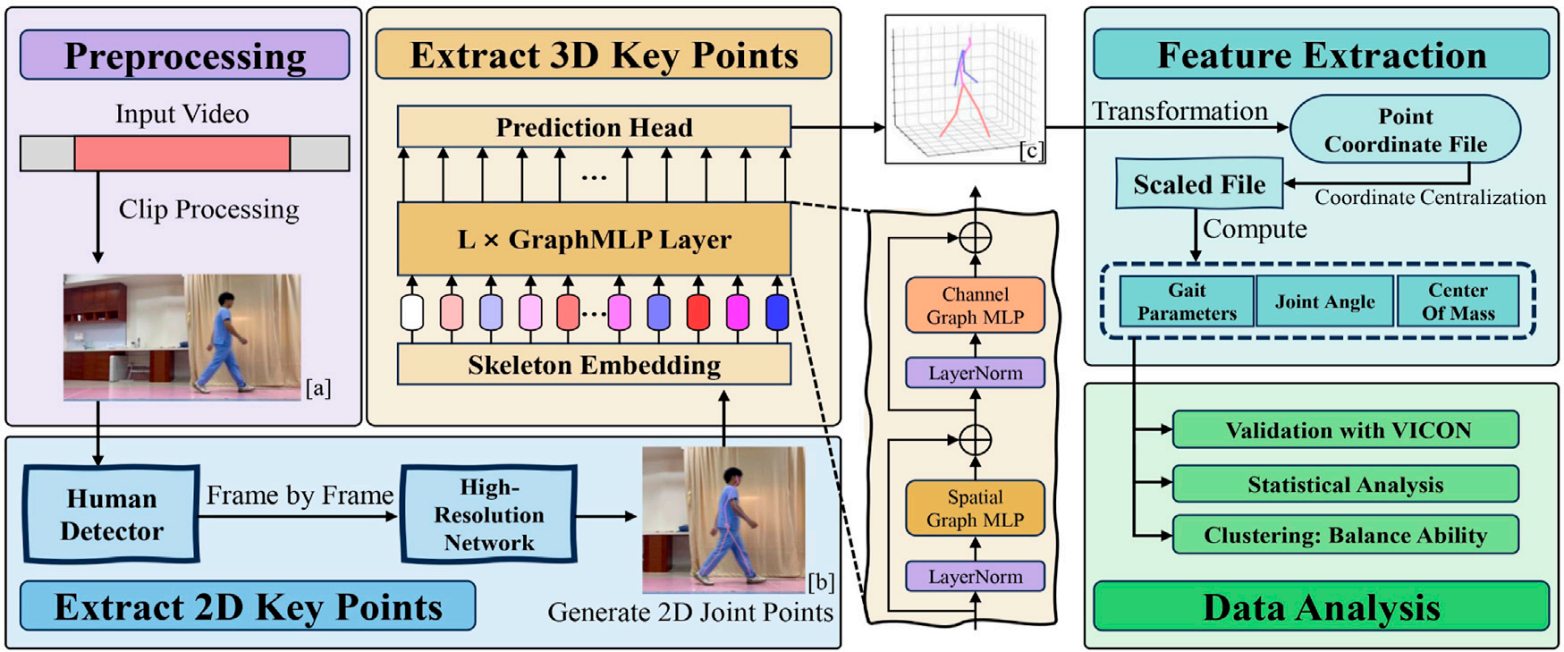
The Flowchart of the Method includes Preprocessing, Extracting 2D key points, Extracting 3D key points, Feature extraction, and Data analysis.

After collecting video data with the smartphone, the following editing rules were applied:

• In the sit-up test, the video segments before the start of each test and after the completion of each sit-up test were trimmed. (exporting 4 data segments).
• In the TUG test, within the retained video data, segments were selected where participants approached the chair from a step away, turned to sit down, then stood up and took a step forward. (from the end of the previous test to the beginning of the next test) (exporting 50 data segments).
• In the gait tests conducted during the validation and measurement experiments, any parts of the video where the participant’s entire body was not fully visible in the frame were edited. (Figure 3a) (exporting 8 data segments for the validation experiment and 100 data segments for the measurement experiment).

### 3.2 Extract joint points

#### 3.2.1 Extracting 2D key points

The High-Resolution Network (HRNet) will be employed to extract precise 2D keypoint information from the video data (Sun et al., 2019). HRNet is a high-resolution convolutional neural network specially designed for processing image data with rich details and multi-scale features, so it is often selected as the 2D key point input model in many 3D key point detection research. To conveniently and fairly compare the experimental results, our research also selected this network as the 2D key point detection model.

Initially, the OpenCV (Bradski, 2000) library is used to obtain the width and height information of the video data, and frames are extracted frame by frame. To ensure the presence of a human body in each frame, the initial frame of the video undergoes target detection.

Subsequently, the HRNet model is employed to extract features from the detected human images, and key point detection is performed on the features for each frame. The output includes each key point’s coordinates and their corresponding confidence scores.

Finally, the data is transformed and corrected, resulting in the output of 2D key point-based human pose estimation (Figure 3b).

#### 3.2.2 Extracting 3D key points

After obtaining the 2D key points, an architecture known as Graph MLP-Like will be utilized to estimate the three-dimensional human pose (Li et al., 2022). GraphMLP is a simple yet effective graph-enhanced multi-layer perceptron (MLP-Like) architecture that combines MLP and Graph Convolutional Networks, enabling local and global spatial interactions to capture more comprehensive information.

Initially, GraphMLP treats each 2D key point as an input token and linearly embeds each key point through skeleton embedding. The input 2D pose, $P\in R^{N\times 2}$ , contains *N* body joints, each regarded as an input token. These tokens are projected through a linear layer into high-dimensional token features $X_{0}\in R^{N\times C}$ , where *C* represents the hidden layer size.

Subsequently, the embedded tokens are passed through the GraphMLP layer, which is characterized by utilizing Graph Convolutional Networks for local feature communication. Each GraphMLP layer consists of a spatial graph MLP (SG-MLP) and a channel graph MLP (CG-MLP). Formulas 1, 2 describe how the MLP layers are modified to handle tokens:

$$X_{l}^{\prime }=X_{l-1}+\text{SpatialMLP}{\left(\text{LN}\left(X_{l-1}^{T}\right)\right)}^{T}+\text{GCN}{\left(\text{LN}\left(X_{l-1}^{T}\right)\right)}^{T}$$ (1)

$$X_{l}=X_{l}^{\prime }+\text{ChannelMLP}\left(\text{LN}\left(X_{l}^{\prime }\right)\right)+\text{GCN}\left(\text{LN}\left(X_{l}^{\prime }\right)\right)$$ (2)

Here, GCN(⋅) represents the GCN block, and *l* ∈ [1, …, *L*] denotes the index of the GraphMLP layer. Here, $X_{l}^{\prime }$ and *X*_*l*_ are the output features of the SG-MLP and CG-MLP for the *l*th block, respectively.

Finally, the prediction head employs a linear layer for regression. It operates on the features $X_{L}\in R^{\left(N\times C\right)}$ extracted from the last GraphMLP layer to predict the final three-dimensional pose $\tilde{X}\in R^{\left(N\times 3\right)}$ . The output is presented as a file containing 3D coordinates of the key points (Figure 3c).

### 3.3 Data postprocessing

After acquiring the 3D key point coordinate file, coordinate centering is applied. This involves subtracting the 3D coordinates of the sacrum point from the 3D coordinates of all key points, resulting in a centered key point coordinate file.

Subsequently, all coordinate points are scaled for further feature extraction. In this study, the lengths of the left and right lower legs, denoted as *L*_left_ and *L*_right_, were measured and recorded for each participant. During the scaling process, for all key points’ coordinates *P*_*i*_ in the *i*th frame, the lengths of the left and right lower legs are calculated using the key points of the left and right knees (*K*_*l*_ and *K*_*r*_) and the left and right ankles (*F*_*l*_ and *F*_*r*_) for that frame. The scaling ratio is then computed and applied to scale all key points:

$${\vec{P}}_{{\text{scaled}}_{i}}={\vec{P}}_{i}\cdot \frac{L_{\text{left}}+L_{\text{right}}}{\left\|\overset{\to }{K_{l}F_{l}}\|+\|\overset{\to }{K_{r}F_{r}}\right\|}$$ (3)

### 3.4 3D pose feature extraction

#### 3.4.1 Knee angles

The scaled file provides the coordinates of the hip joint *H*_*i*_, the knee joint *K*_*i*_, and the ankle joint *A*_*i*_ for that frame. The knee joint angles for the *i*th frame are determined using the Formula 4:

$$\theta_{i}=\cos^{-1}\left(\frac{\left(\overset{\to }{H_{i}K_{i}}\right)\cdot \left(\overset{\to }{A_{i}K_{i}}\right)}{\left\|\overset{\to }{H_{i}K_{i}}\|\cdot \|\overset{\to }{A_{i}K_{i}}\right\|}\right)$$ (4)

#### 3.4.2 Knee angular velocity

Given the frame rate of the video data as *f* (30 Hz), after calculating the knee joint angle for the *i*th frame, the knee angular velocity for the *i*th frame can be determined using Formula 5:

$$\omega_{i}=f\cdot \left(\theta_{i}-\theta_{i-1}\right)$$ (5)

#### 3.4.3 Step lengths

Obtain the coordinates of the left and right ankle joints, denoted as *A*_*l*_ *i* and *A*_*r*_ *i*, from the scaled file. Then, compute the distance *D*_*i*_ between the ankle joints of the left and right legs for the *i*th frame:

$$D_{i}=\|\overset{\to }{A_{li}A_{ri}}\|$$ (6)

Subsequently, filter all local maxima based on the threshold to obtain the step length *S*.

#### 3.4.4 Step side

For the *i*th step length *S*_*i*_ and its time *T*_*i*_: Obtain the coordinates of the sacral vertebrae point $S_{V_{i}}$ , thoracic vertebrae point $T_{V_{i}}$ , and cervical vertebrae point $C_{V_{i}}$ at the time *T*_*i*_. Use the coordinates of these three points to calculate the sagittal plane *Sa*_*i*_ at the time *T*_*i*_ and use Formula 7 to calculate the coronal plane *Co*_*i*_ at the time *T*_*i*_:

$${Co}_{i}⊥{Sa}_{i},\;{Co}_{i}⊥\left(\overset{\to }{S_{V_{i}}C_{V_{i}}}\right)$$ (7)

Subsequently, calculate the distances from the left and right ankle joint coordinates *A*_*li*_ and *A*_*ri*_ to the coronal plane *Co*_*i*_. Determine the relative position of both feet to the body at time *T*_*i*_ (step side).

#### 3.4.5 Step period

After obtaining the step side, the step period *p* corresponding to each step side can be calculated using the step length’s corresponding time *T*:

$$p_{i}=T_{i}-T_{i-1}$$ (8)

#### 3.4.6 Walking speed

The walking speed *v* corresponding to each step side can be calculated using the step length *S* and step period *p*:

$$v_{i}=\frac{S_{i}+S_{i-1}}{2p_{i}}$$ (9)

#### 3.4.7 Step phase

First, calculate all local maxima *ω*_max_ and all local minima *ω*_min_ for knee joint angular velocity *ω*. Sort all *ω* values and use the positions corresponding to the 20th and 80th percentiles as thresholds. Remove extreme values that do not exceed these thresholds.

Next, set the time corresponding to *ω*_min_ as the moment when that foot separates from the ground (toe-split), denoted as *t*_split_. Starting from *ω*_max_, search along the time direction for the first instance when *ω* falls below ten degrees/s and note the corresponding time as *t*_touch_, representing the moment when that foot makes contact with the ground (heel-strike).

Finally, calculate the swing phase time *t*_swing_ and support phase time *t*_support_ as follows:

$$t_{{\text{swing}}_{i}}=t_{{\text{touch}}_{i}}-t_{{\text{split}}_{i-1}}$$ (10)

$$t_{{\text{support}}_{i}}=t_{{\text{split}}_{i}}-t_{{\text{touch}}_{i-1}}$$ (11)

#### 3.4.8 Center of mass

Using a kinematic approach and an 11-segment body model, calculate the COM of the entire body. Firstly, exclude the lighter parts, which are the hands and feet, and divide the rest of the body into 11 segments: head, upper trunk, lower trunk, two upper arms, two forearms, two thighs, and two lower legs (Yang et al., 2014).

Next, based on anthropometric data for the Chinese population 17245–2004 (SPC, 2004), calculate the weight of each segment and use the extracted 3D coordinates of 17 key points to compute the COM of the 11 segments. Finally, calculate the whole-body COM by weighting the relative positions of all body segments Formula 12.

Since the key point coordinates have been subjected to coordinate centering, the COM obtained here is relative to the body’s coordinate system. In the validation experiment, the relative COM position is calculated with respect to the left foot as the reference point since the Vicon markers on the left foot align well with the foot key points extracted from the smartphone data. In the measurement experiment, the relative COM position is calculated with respect to the hip region as the reference point.

$$\text{COM}=\frac{1}{N}\overset{N}{\underset{i=0}{\sum }}\frac{{\text{COM}}_{i}\cdot m_{i}}{m_{w}}$$ (12)

Where *N* is the total number of segments, which in this study is 11, COM_*i*_ represents the center position coordinates of the *i*th segment, *m*_*w*_ is the total body mass, and *m*_*i*_ is the mass of the *i*th segment.

Using the COM_*a*_ of the *a*th frame, as well as the sagittal plane *Sa*_*a*_ and coronal plane *Co*_*a*_, the deviation distances of the COM from both the coronal and sagittal planes for the *a*th frame can be determined. By performing these calculations for all frames, the range of deviation of the COM from both the coronal and sagittal planes can be obtained.

#### 3.4.9 Relative center of mass velocity

To better compare the participant’s self-balance ability during various tasks, the RCOMV will be calculated as follows:

$$\text{RCOMV}=f\cdot \left({\text{COM}}_{a}-{\text{COM}}_{a-1}\right)$$ (13)

Where the frame rate is denoted as *f* = 30Hz, and COM_*a*_ represents the COM for the *a*th frame.

### 3.5 Statistical analysis

For statistical analysis, the key point data collected by Vicon in the validation experiment will be resampled, reducing the data frequency from *f* (100 Hz) to match the data frequency collected by the smartphone, which is *f* (30 Hz). Subsequently, the key point data collected by Vicon and the smartphone will be temporally aligned. The knee joint angles will be used as the alignment feature for the two sets of data, where *X*(*t*) represents the knee joint angle data obtained from Vicon, and *Y*(*t*) represents the knee joint angle data obtained from the smartphone:

$$C\left(\tau \right)=\int_{-\infty }^{\infty }X\left(t\right)Y\left(t+\tau \right)\;dt$$ (14)

Where *t* represents time, *τ* represents a time shift, and by calculating the cross-correlation function *C*(*τ*), the time shift *τ*_max_ that maximizes the cross-correlation value is obtained. Subsequently, the data is temporally shifted to achieve optimal time-domain alignment between the two datasets.

Intraclass correlation coefficients (ICC(2,k)) are used to evaluate all parameters calculated from data obtained from Vicon and the smartphone in the gait test section of the validation experiment. The ICC values are interpreted based on Cicchetti’s guidelines, where ICC <0.40 : poor reliability, 0.40 ≤ ICC <0.60 : fair reliability, 0.60 ≤ ICC <0.75 : good reliability, and ICC ≥0.75 : excellent reliability (Cicchetti, 1994).

Pearson’s correlation coefficient (*r*) is used to examine the degree of correlation between all parameters calculated from data obtained from Vicon and the smartphone in the gait test section of the validation experiment. The correlation strength can be interpreted based on the *r* values as follows: *r* < 0.30: Negligible correlation, 0.30 ≤ *r* < 0.50: Low correlation, 0.50 ≤ *r* < 0.70: Moderate correlation, 0.70 ≤ *r* < 0.90: High correlation, 0.90 ≤ *r* ≤ 1.00: Very high correlation (Mukaka, 2012).

To select suitable features for better clustering results between patients and healthy individuals in the measurement experiment’s gait test section, Welch’s *t*-test (Welch, 1938) is used to assess the differences in all parameters calculated from Phone data between patients and healthy individuals. The significance level is set to *p* < 0.05, following Prescott’s statistical guidelines (Prescott, 2019).

All data are analyzed using Python.

## 4 Results

### 4.1 Reliability and validity verification

In terms of reliability, all results for the parameters computed from data collected from both Vicon and Phone demonstrate excellent reliability (Vicon, ICC (2,k): 0.853–0.982; Phone, ICC (2,k): 0.839–0.975). (Table 2).

**TABLE 2:** Reliability and validity of obtained parameters in the validation experiment.

Parameter	VICON		Phone		
	Mean(SD)	ICC(2,k)	Mean(SD)	ICC(2,k)	r
Average step period(s)	0.71 (0.05)	0.946	0.73 (0.06)	0.965	0.898
Average walking speed (mm/s)	753 (68.7)	0.918	696 (68.9)	0.898	0.942
Right knee angle ROM(°)	55.8 (6.46)	0.982	56.0 (3.87)	0.937	0.953
Left knee angle ROM(°)	55.2 (9.38)	0.957	55.6 (6.74)	0.927	0.973
Right knee angular velocity range (°/s)	518 (53.7)	0.942	525 (42.9)	0.975	0.808
Left knee angular velocity range (°/s)	482 (103)	0.947	490 (100)	0.843	0.934
Average relative center of mass velocity (mm/s)	906 (111)	0.853	899 (127)	0.839	0.978

In terms of validity, a comparison between the parameters calculated from Phone and Vicon data revealed that all parameters showed a high to very high level of correlation (*r*: 0.808–0.978, *p* < 0.05).

**FIGURE 4:**
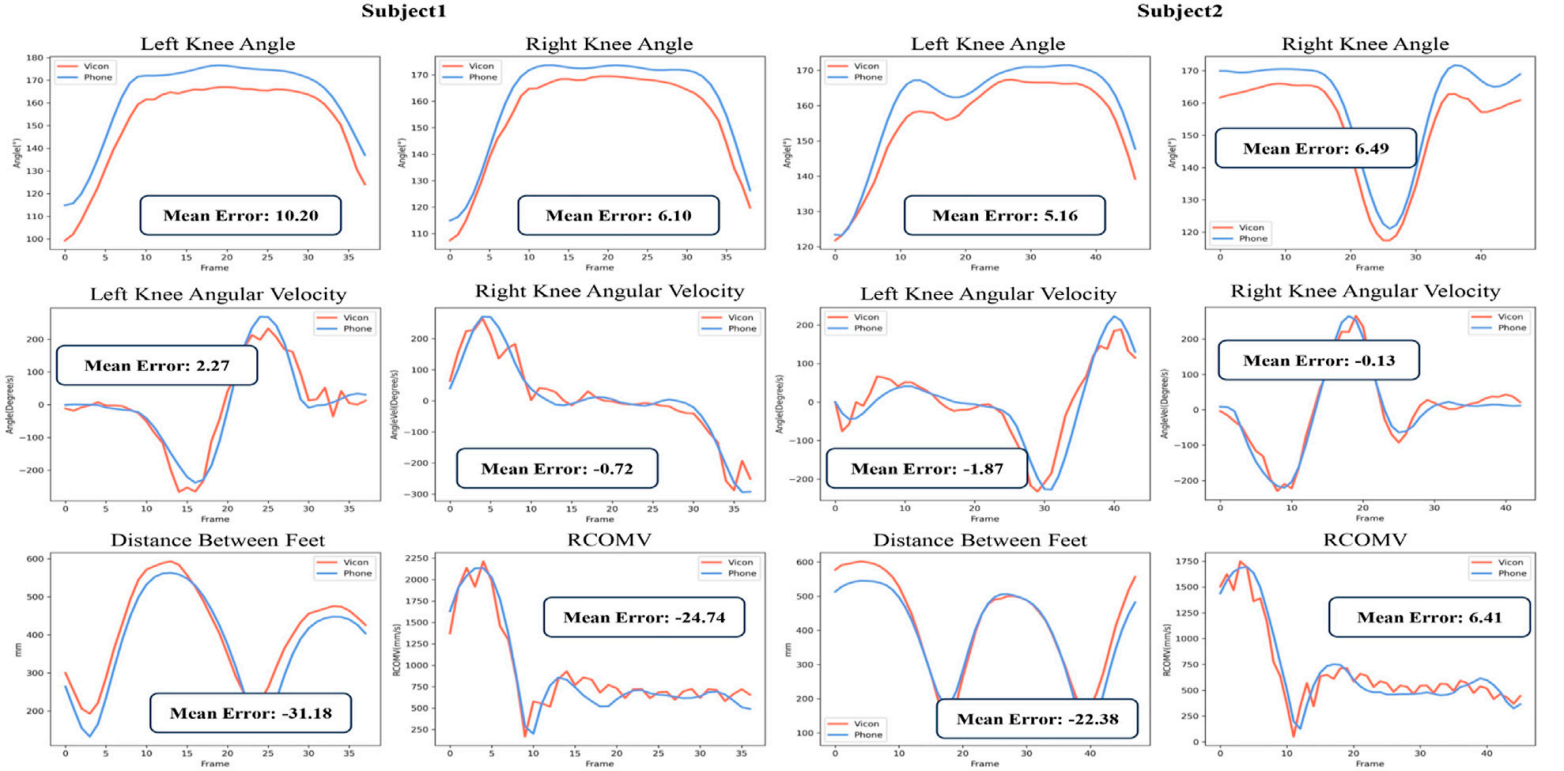
Partial Parameter Results in the Validation Experiment:Vicon data (red) and Phone data (blue).

Figure 4 displays the knee angles, angular velocities, the distance between the feet, and RCOMV measured during the validation experiment. From the figure, it is evident that there is a significant correlation between the results obtained from Phone and Vicon. In the measurement of knee angles, the results obtained by Phone were consistently slightly higher than those obtained by Vicon, while the differences in others were relatively small.

### 4.2 Gait parameter results

**FIGURE 5:**
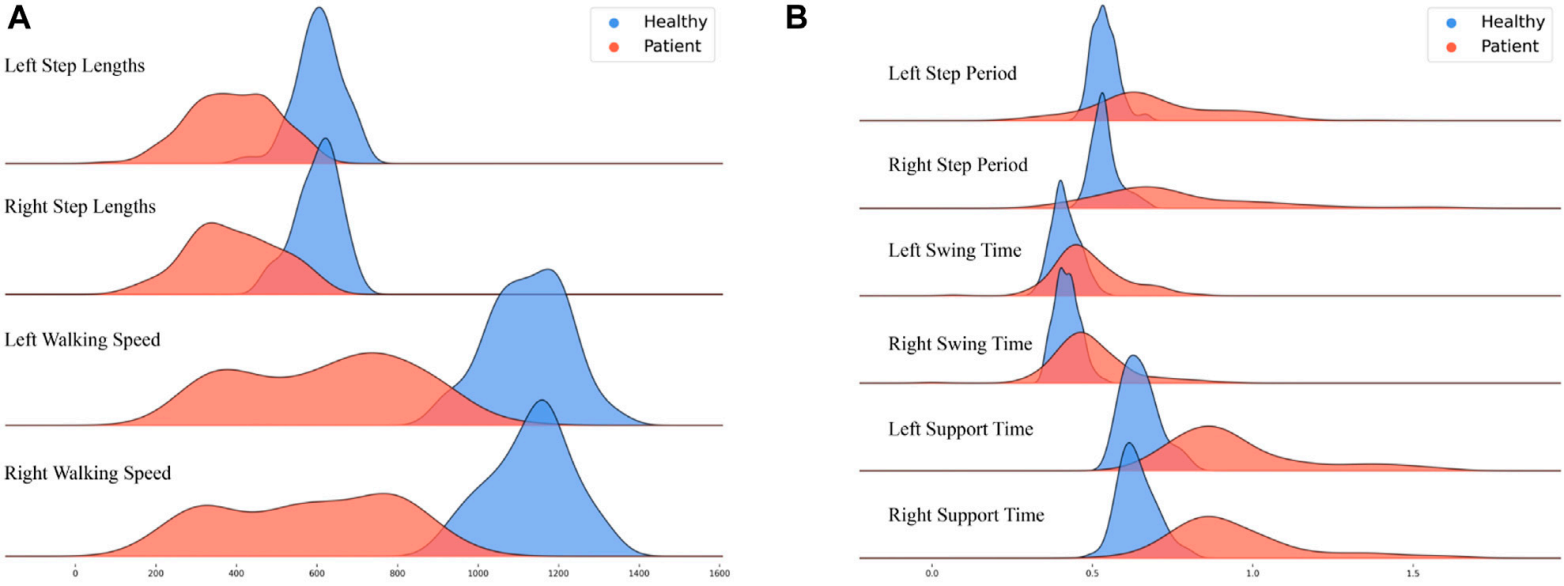
Distribution of Gait Spatiotemporal Parameter Results: Healthy adults (blue) and patients (red); **(A)** Left step lengths, Right step lengths, Left walking speed, and Right walking speed; **(B)** Left step period, Right step period, Left swing time, Right swing time, Left support time, and Right support time.

Table 3 presents the gait parameter results for each participant, including step lengths, walking speeds, and swing/support times for each foot. Figure 5 displays the spatiotemporal distribution of gait parameters.

**TABLE 3:** Gait parameter results.

Subject	Mean(SD)						Swing time/Support time	
	Left step lengths(mm)	Right step lengths(mm)	Left step period(s)	Right step period(s)	Left walking speed(mm/s)	Right walking speed(mm/s)	Left step phrase(s/s)	Right step phrase(s/s)
1	627 (26)	611 (39)	0.64 (0.03)	0.64 (0.02)	969 (70)	974 (43)	0.51/0.77	0.48/0.78
2	686 (29)	641 (40)	0.57 (0.02)	0.57 (0.03)	1165 (74)	1197 (82)	0.45/0.68	0.46/0.67
3	661 (27)	666 (32)	0.57 (0.02)	0.56 (0.02)	1181 (72)	1199 (39)	0.41/0.69	0.42/0.69
4	591 (29)	580 (57)	0.52 (0.02)	0.5 (0.01)	1124 (68)	1180 (87)	0.4/0.61	0.4/0.6
5	585 (30)	592 (38)	0.55 (0.02)	0.55 (0.04)	1074 (87)	1079 (96)	0.4/0.68	0.41/0.67
6	600 (26)	583 (50)	0.52 (0.02)	0.52 (0.02)	1145 (53)	1143 (76)	0.41/0.63	0.42/0.62
7	575 (66)	567 (61)	0.52 (0.02)	0.54 (0.03)	1098 (100)	1069 (80)	0.43/0.62	0.42/0.63
8	657 (46)	640 (41)	0.51 (0.02)	0.53 (0.02)	1276 (59)	1232 (65)	0.4/0.62	0.39/0.62
9	530 (57)	562 (58)	0.53 (0.03)	0.52 (0.02)	1019 (79)	1078 (94)	0.43/0.63	0.43/0.62
10	620 (30)	605 (46)	0.57 (0.02)	0.53 (0)	1075 (67)	1159 (12)	0.43/0.64	0.42/0.69
11	549 (25)	539 (45)	0.49 (0.02)	0.53 (0.01)	1122 (66)	1052 (39)	0.39/0.62	0.43/0.58
12	634 (47)	638 (26)	0.54 (0.01)	0.54 (0.02)	1194 (20)	1168 (70)	0.39/0.67	0.44/0.59
13	588 (38)	604 (32)	0.5 (0)	0.49 (0.02)	1199 (39)	1229 (66)	0.38/0.59	0.37/0.59
14	264 (57)	349 (54)	0.8 (0.2)	0.9 (0.2)	422 (125)	358 (102)	0.55/1.1	0.62/1.1
15	461 (26)	454 (50)	0.63 (0.05)	0.61 (0.04)	740 (77)	758 (74)	0.45/0.75	0.48/0.75
16	400 (68)	380 (71)	0.61 (0.14)	0.69 (0.11)	674 (125)	599 (116)	0.41/0.89	0.44/0.86
17	352 (94)	255 (82)	0.7 (0.28)	0.64 (0.18)	536 (253)	508 (155)	0.49/0.83	0.43/0.89
18	497 (104)	553 (45)	0.67 (0.06)	0.65 (0.06)	825 (85)	819 (75)	0.47/0.87	0.4/0.91
19	487 (95)	516 (94)	0.66 (0.06)	0.66 (0.09)	792 (118)	782 (69)	0.49/0.85	0.43/0.81
20	538 (60)	501 (66)	0.67 (0.06)	0.67 (0.04)	800 (78)	785 (85)	0.46/0.85	0.47/0.85
21	345 (62)	314 (52)	0.69 (0.28)	0.81 (0.3)	573 (224)	493 (232)	0.44/1	0.49/0.98
22	299 (52)	326 (33)	0.88 (0.22)	1.14 (0.34)	384 (105)	304 (110)	0.67/1.43	0.67/1.4
23	481 (49)	526 (65)	0.57 (0.03)	0.61 (0.06)	900 (97)	855 (118)	0.41/0.74	0.4/0.76
24	450 (41)	457 (56)	0.67 (0.11)	0.73 (0.07)	698 (130)	632 (79)	0.53/0.84	0.52/0.86
25	366 (28)	392 (67)	0.66 (0.05)	0.65 (0.11)	589 (58)	597 (133)	0.44/0.91	0.48/0.87

The figures show that healthy adults’ results are more concentrated than those of the patients. Regarding gait spatial parameters, the results for healthy adults are greater than those for the patients (Figure 5A). Conversely, regarding gait temporal parameters, the results for healthy adults are generally smaller than those for the patients (Figure 5B).

The difference between healthy adults and patients is relatively minimal regarding swing time for the left and right feet (with a larger overlap in peak areas).

### 4.3 Knee angle results

**FIGURE 6:**
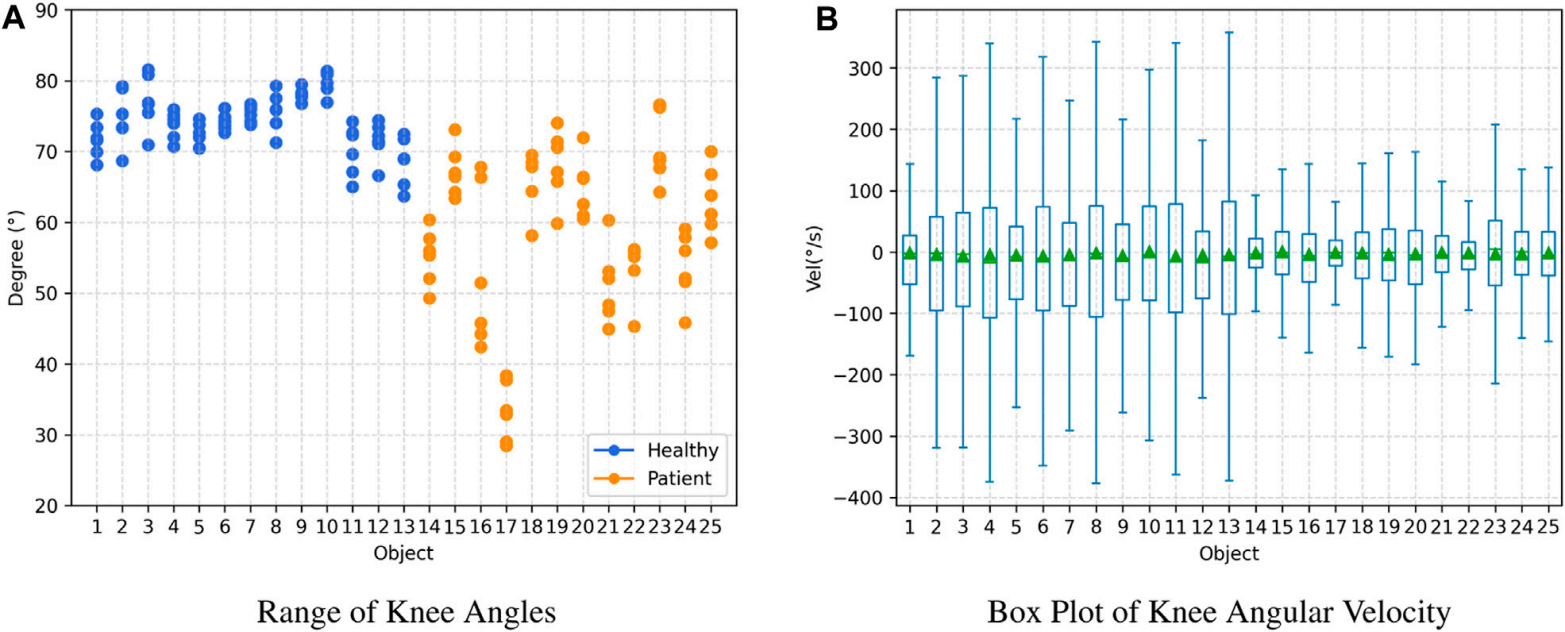
Joint Angle Results: Healthy adults (subject 1–13) and patients (subject 14–25). **(A)** Range of Knee Angles, **(B)** Box Plot of Knee Angular Velocity.

Figure 6 depicts the range of knee joint angles and knee joint angular velocities observed among participants during the gait testing in the observational experiment. Overall, healthy adults’ knee joint angle and angular velocity range are more extensive than those for patients. In the knee joint angle range plot, data for healthy adults are relatively concentrated, with values consistently above 60°. In contrast, data for patients are distributed within the range of 30°–80°. Within the knee joint angular velocity range plot, healthy adults generally exhibit values above 300, while patients’ data mostly fall below 300.

### 4.4 Center of mass results

**FIGURE 7:**
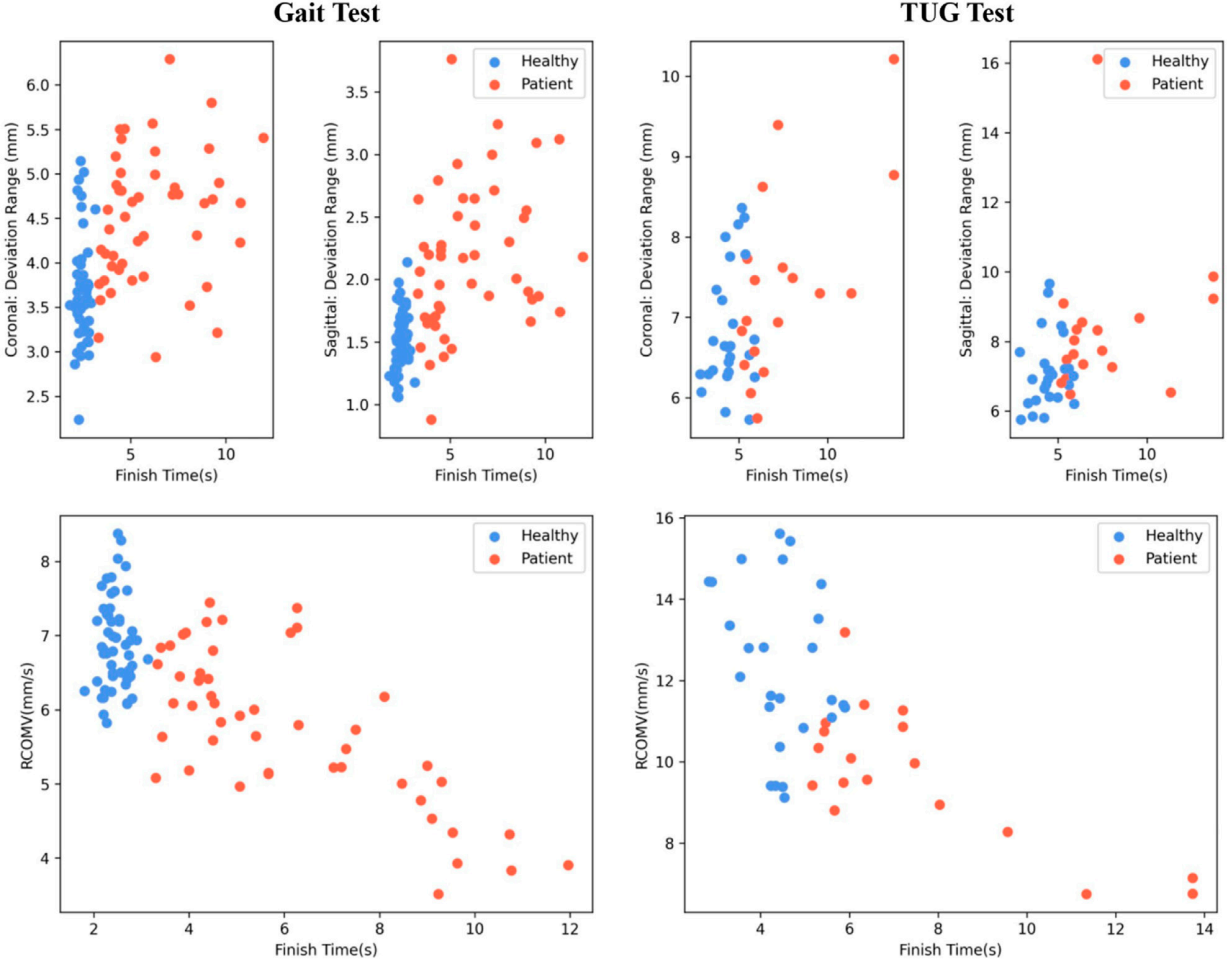
COM Results: Healthy adults (blue) and patients (red).

Figure 7 illustrates the RCOMV, the deviation range from the sagittal and coronal planes during gait and TUG testing in the measurement experiment. The horizontal axis represents the time taken to complete the tests.

Overall, healthy adults complete the tests faster than patients. The RCOMV for healthy adults during gait and TUG testing is greater than that for patients. In gait testing, the deviation range from the coronal and sagittal planes for healthy adults is slightly lower than that for patients. However, in TUG testing, the deviation range from the coronal and sagittal planes for healthy adults shows little difference compared to that of patients.

### 4.5 Cluster results

Table 4 displays the differences in characteristics between patients and healthy individuals in the measurement experiment. The *t*-test is used to determine the differences in means between healthy individuals and patients for each feature. All parameters obtained from healthy individuals and patients exhibit significant differences (*p* < 0.01). Therefore, the features in the table are suitable as input features for cluster analysis to distinguish different levels of balance ability.

**TABLE 4:** Differences in characteristics between patients and healthy individuals in the measurement experiment.

Parameter	Mean (SD)		t	p
	Healthy adults	Patients		
Average step period(s)	0.54 (0.04)	0.71 (0.12)	−9.42	<0.01
Average step length (mm)	605 (42.2)	416 (85.9)	13.85	<0.01
Average walking speed (mm/s)	1131 (81.4)	644 (169)	18.1	<0.01
Right knee angle ROM(°)	74.2 (4.36)	58.5 (11.1)	9.19	<0.01
Left knee angle ROM(°)	73.5 (5.47)	58.1 (12.6)	7.81	<0.01
Right knee angular velocity range (°/s)	808 (87.2)	554 (154)	10.1	<0.01
Left knee angular velocity range (°/s)	808 (89.8)	585 (173)	7.99	<0.01
Average relative center of mass velocity (mm/s)	6.90 (0.62)	5.77 (1.03)	6.62	<0.01
Deviation range from COM to sagittal plane (mm)	1.52 (0.24)	2.15 (0.58)	−7.11	<0.01
Deviation range from COM to coronal plane (mm)	3.69 (0.59)	4.51 (0.73)	−6.12	<0.01

**FIGURE 8:**
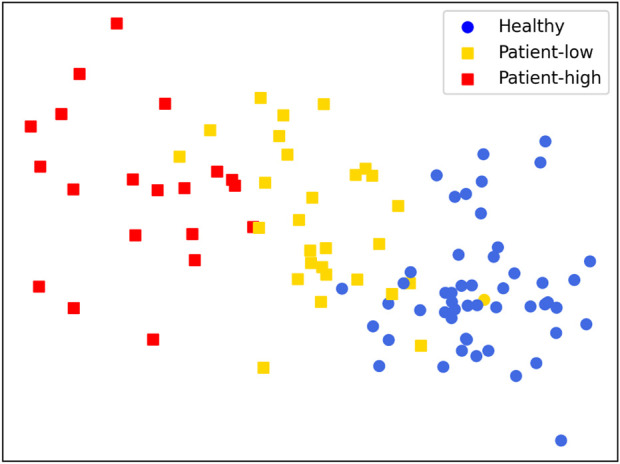
Clustering Results:Healthy adults (circles), patients (squares).

Figure 8 presents the results of clustering the gait test data of 25 participants (4 data points per person, totaling 100 data points). All parameters calculated from the gait test data collected via Phone were used as inputs. The K-Means++ clustering algorithm was employed with clusters set to 3 to reveal potential patterns within the data (Arthur and Vassilvitskii, 2007).

Overall, the clustering results are significant. Although one data point from a healthy adult participant was incorrectly classified into the patient group, the remaining data were successfully grouped. Meanwhile, the data points for the patient group were well clustered into two distinct groups: high and low levels of rehabilitation progress or balance ability. This classification helps guide and personalize the patient’s ability to improve balance control.

## 5 Discussion

The purpose of this study is to explore the reliability and effectiveness of 3D human pose estimation based on smartphone monocular video, as well as the feasibility of human balance ability assessment. We propose a novel simple and effective HGcnMLP algorithm for markerless 3D pose estimation and validate it on 15 healthy adults and 12 patients with musculoskeletal diseases. The main findings and contributions are: a) The human gait parameters, knee angle parameters, and RCOMV estimated based on the HGcnMLP algorithm show high reliability, and show excellent consistency with VICON gold standard results; b) The 3D pose estimation method based on smartphone monocular video has the potential to evaluate the gait balance ability of healthy adults and patients, and is expected to solve the problem of lack of simple, effective and easy-to-operate systems in the field of medical diagnosis/rehabilitation evaluation; c) It is the first motion capture method based on the monocular video to be used in research on human biomechanics. It is a useful attempt at the clinical application of this technology and promotes the possibility of emerging computer vision technology in clinical auxiliary diagnosis and remote rehabilitation evaluation.

### 5.1 Reliability and validity of parameter estimates

Most of the parameters based on VICON and Phone in this study show very high reliability (ICC (2, k): 0.839–0.982), which is better than our previous research based on the OpenPose algorithm framework (ICC: 0.506–0.734) (Liang et al., 2022). Azhand et al. proposed a highly efficient and reliable gait parameter estimation algorithm based on monocular video, and all measured gait parameters showed excellent intra-class correlation coefficient results (ICC (2, k): 0.958 and 0.987), comparable to ours (Azhand et al., 2021). In addition, compared with the VICON motion capture system, all extraction parameters in this study also showed high to very high effectiveness (Pearson r: 0.808–0.978, *p* < 0.05), consistent with the latest research results (r: 0.921) (Srinivasan et al., 2023). Although the actual interference of participants’ height/weight on the accuracy of mobile phone and Vicon measurements is unclear, we still tried to make the height/weight difference of participants as small as possible to reduce the impact on reliability (Table 1, height *p* = 0.04, weight *p* = 0.25). The average step period error is 0.02s, the average left-right knee angle ROM error is about 0.3°, and the knee angular velocity and center of mass velocity are both ≤8°/s (mm/s). We also noticed that the SD value of the angular velocity range of the left knee is higher than that of the right knee. It is speculated that in the experiment we designed, the right leg covers the left leg more than the left leg covers the right leg, resulting in relatively unstable key points extraction on the occluded left side. Nonetheless, the above observations provide preliminary evidence that 3D pose estimation based on smartphone monocular video is suitable for objectively measuring parameters such as gait time, joint angles, and center-of-mass velocity of subjects.

Shin et al. performed quantitative gait analysis on a single 2D image with a pose estimation algorithm based on deep learning. The average error of walking speed was 8.95 cm/s, while our average error result was 5.7 cm/s, showing better performance (Shin et al., 2021). Steinert et al. used a smartphone camera and a deep convolutional neural network to analyze the gait of the elderly on a 3D skeletal model. The gait parameters showed comparable accuracy to ours, but the reliability of parameter estimation needs to be further improved (ICC (1, 1): 0.125–0.535) (Steinert et al., 2019). Stenum et al. compared the spatiotemporal and sagittal motion gait parameters measured using OpenPose (an open-source video-based human pose estimation) with simultaneously recorded 3D motion capture results, showing that the average absolute error in step time and knee angle (step time: 0.02 s/step; knee angle: 5.6°) was comparable to ours (Stenum et al., 2021b). Gu et al. calculated the accuracy of knee angles of the lower limbs based on the OpenPose model by using a single mobile phone to track the joint coordinates of healthy adults during walking, and the results showed an error of 10° Gu et al. (2018). Hellsten et al., 2021 reported knee angle errors of 10° or less in most tracked frames, the accuracy comparable to marker-based camera systems. In addition, our angular velocity and center-of-mass velocity achieved excellent results (≤8°/s (mm/s)) compared to the gold standard, which has important implications for patient balance assessment. We acknowledge that some of the latest Transformer-based monocular models can improve 2D poses to 3D poses with smaller joint position errors than this system. For example, the MotionBERT model (Zhu et al., 2023) and the Graph-based GLA-GCN network model (Yu et al., 2023), etc. Relatively speaking, the GraphMLP model is more lightweight, has fewer parameters, has low computational cost, and is more suitable for our pursuit of low-cost and simple clinical goals. Overall, the HGcnMLP estimation method proposed in this study can improve the accuracy of human posture estimation in monocular videos, and has good reliability and validity, which meets the clinical requirements for system performance.

### 5.2 Comparison of healthy adult and patient pose parameters

To compare the gait balance ability of healthy adults and patients with sarcopenia (osteoarthritis), the TUG gait test was performed, and gait parameters, joint angle parameters, and center-of-mass velocity were extracted (Åberg et al., 2021). The spatiotemporal parameters in healthy adults showed faster speed, larger step length, and more centralization, whereas patients showed longer step and support/swing times with a wider distribution, suggesting a difference between the two groups (Figure 5; Table 3). In addition, the knee joint angle of healthy adults showed a larger range of motion ( > 60°), while patients included three levels: 30°–40°, 40°–60°, and ≥60°, indicating patients tend to slow flexion and extension during walking, this is detrimental in dealing with some acute events (Figure 6). This study also estimated the participant’s relative COM velocities and ranges of COM offset from the sagittal/coronal plane, which usually requires the help of some 3D motion capture systems (e.g., Optotrak Certus and VICON) based on markerless monocular video motion capture (Yang et al., 2014). Healthy adults showed greater COM velocities during TUG testing, and a slightly lower range of COM offsets from the coronal and sagittal planes than patients, indicating better gait stability (Figure 7).

Shin et al. performed quantitative gait analysis on 2D videos of Parkinson’s patients based on a pose estimation algorithm and demonstrated that the proposed method can objectively estimate gait parameters (Shin et al., 2021). Kim et al., 2023 assessed the physical abilities of sarcopenic patients using smartphone video pose estimation and smart insoles to develop patient digital biomarkers. Azhand et al. demonstrated the effectiveness of a monocular smartphone video-based 3D pose estimation algorithm for gait assessment applications in the elderly relative to the gold-standard gait assessment system GAITRite (Azhand et al., 2021). Current research mainly focuses on simple analysis of gait parameters (gait speed, frequency, step length, and step time), but rarely analyzes related parameters such as joint angles and COM velocity (Gu et al., 2018). This study compared three groups of pose features between patients and healthy people, all showing differences (Table 4, p < 0.01), which implies that the extracted features can be used for balance and rehabilitation assessment. To further evaluate the performance of the proposed system in human gait balance level and rehabilitation progress, the K-Mean++ clustering algorithm was used to successfully distinguish patients into different groups and achieved consistent results with professional physical therapist scale evaluations (Figure 8). In summary, this work demonstrates the potential of single smartphone 3D human pose estimation for clinical motion capture, promising comprehensive assessment of balance abilities between healthy and patients.

### 5.3 Research limitations and future work

We acknowledge that this study has some limitations. First, we only performed data collection at 90° in the walking direction, although previous work has shown that this direction is most conducive to pose estimation (Rui et al., 2023), and more positions can be used to record richer motion poses. In addition, the number of participants was relatively small, and there was no elderly normal subject group, which may affect the reliability of the study. In clustering patients’ balance ability, although the results were consistent with clinical scale evaluations, manual classification of the clustering results was considered a limitation. Finally, the current smartphone video data relies on offline processing by laptop computers and cannot automate this preprocessing by detecting critical time points, which limits clinical and home rehabilitation applications.

In the future, we need to expand the number of participants to further verify and improve the reliability of the results. Of course, we admit that more advanced and accurate 2D key point extraction models will achieve better results, and the latest 2D key point extraction models will be considered in subsequent clinical experiments. Furthermore, we need to develop a novel smartphone application for 3D pose estimation, perform human motion capture directly from camera 2D images, and develop an application interface to visualize parameters and provide reliability and validity results.

## 6 Conclusion

In this study, we preliminarily observed that the markerless 3D pose estimation method based on smartphone monocular video can provide effective and reliable human pose parameters, and can provide good accuracy for the rehabilitation evaluation of patients with musculoskeletal diseases. Although in the field of computer vision algorithms, the performance of the proposed method can be further improved, this work is a useful attempt to use single-view technology in clinical disease diagnosis and balance evaluation and will help promote the application of emerging computer vision technology in the medical field. Given the advantages of the proposed model such as simplicity, low cost, and portability, the markerless 3D pose estimation system is expected to provide a clinical alternative to human motion capture and provide a new easy-to-operate tool for remote monitoring and home rehabilitation assessment.

## Data Availability

The raw data supporting the conclusion of this article will be made available by the authors, without undue reservation.
